# Improvements to a framework for gender and emerging infectious diseases

**DOI:** 10.2471/BLT.20.275636

**Published:** 2021-07-02

**Authors:** Lynn Lieberman Lawry, Roberta Lugo-Robles, Vicki McIver

**Affiliations:** a Uniformed Services University of the Health Sciences, Preventive Medicine and Biostatistics Department, 4301 Jones Bridge Rd, Bethesda, MD 20814-4799, United States of America (USA).; b 11032 Peach Ct, Belle Center, Logan, USA.

Sex and gender issues are important during pandemics and epidemics; however, they are routinely overlooked. In emerging infectious disease contexts, sex and gender factors affect the vulnerability, exposure risk, treatment and response that may affect the incidence, duration, severity, morbidity, mortality and disability of those who become infected.^1^

Even before the coronavirus disease 2019 (COVID-19) pandemic, studies showed that the gendered nature of health-care settings, roles in society and power structures have a role in emerging infectious disease outcomes.^1^^,^^2^ In the World Health Organization’s analytical framework on sex and gender in emerging infectious diseases, sex differences focus on pregnancy, anatomical and immunological differences; gender is mainly associated with outcomes related to disease prevention and control programmes (Fig. 1).^1^ By focusing on these limited differences, the framework does not consider the full spectrum of sex and more inclusive gender effects on health outcomes in emerging infectious diseases. 

**Fig. 1:**
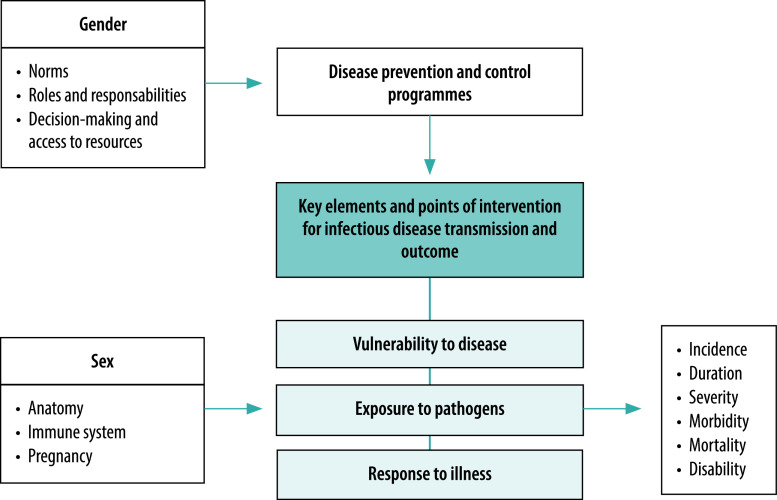
Sex, gender and emerging infectious disease framework

Source: World Health Organization, 2011.^1^

The lessons learnt from the COVID-19 pandemic and other emerging infectious diseases epidemics, such as the Ebola virus disease, Zika virus, Middle East respiratory syndrome and severe acute respiratory syndrome (SARS), reveal that current frameworks on the effects of sex and gender have gaps. These shortages should be addressed to improve strategic planning and public health response to emerging infectious disease pandemics and/or epidemics, based on needs of males, females, intersex, cisgender, transgender and non-binary persons.^1^^,^^2^ An ideal emerging infectious disease framework for sex and gender should include a full spectrum of sex and gender factors that have influence on disease vulnerability, whether direct (through the exposures to infectious pathogens and responses to illness) or indirect (through effects on disease prevention and control programmes).^1^^,^^2^

## Sex-based gaps

Physiological and biological factors define males, females and intersex and include chromosomal, hormonal and anatomical characteristics.^2^ Hormonal differences, pharmacokinetics and pharmacodynamics are rarely considered factors. A full understanding of the vertical and horizontal transmission of emerging infectious diseases is important and should be systematically considered early during outbreaks.

Sex steroid hormones, such as estrogen, can alter gene expression to have a protective role, and account for females’ ability to mount a more vigorous immune response to infections and produce greater antibody responses to vaccines – which is a pharmacodynamic factor.^3^ Thus, hormonal status may explain lower fatality rates among females during COVID-19 and better immune response to vaccines.^3^ Hormonal changes and hormonal replacement therapy in menopausal, postmenopausal or gender transitioning individuals or anti-hormonal therapy for reproductive cancers can also lead to altered drug disposition and absorption.^3^

Pharmacokinetic sex differences in body weight, total body water, extracellular and intracellular water, total volume of blood, plasma and red blood cells, hormones, kidney function, hepatic function, gastric emptying and/or intestinal motility and cardiac output affect drug absorption, distribution, metabolism and elimination.^3^ For example, women require less medication for sedation on ventilators than men; these medications have been in short supply during the COVID-19 pandemic, hence the importance of considering pharmacokinetic sex differences.^4^ Finally, renal blood flow in pregnant versus non-pregnant individuals also affects drug pharmacokinetics such as the elimination of some medications.^3^

Vertical and horizontal transmissions are important sex factors that are not addressed early because the focus is generally on pregnant individuals.^5^ Evidence suggests vertical transmission of SARS coronavirus 2 (SARS-CoV-2) takes place when the infection occurs in the third trimester of pregnancy at a rate (pooled proportion) of 3.2% (95% confidence interval, 2.2–4.3) which is similar to that of congenital infection rates among other emerging infectious diseases.^6^ Similar to the Ebola virus, horizontal (that is, sexually transmitted) SARS-CoV-2 has been found in the semen of recovering male patients.^7^ Whether transmission of latent or active virus exists over time remains to be determined; such knowledge would have broad public health implications.^7^

## Gender gaps

Gender is defined as the socially constructed roles, behaviours, activities and attributes that a given society considers appropriate for males and females.^2^ Rarely is the full construct of gender, especially sexual and gender minorities, considered in emerging infectious diseases. COVID-19 reduced access to gender-affirming resources and the ability of transgender and non-binary people to live according to their gender, and increased the rates of mental health disorders, social isolation and violence among transgender and non-binary individuals.^8^

The COVID-19 pandemic triggered extensive and severe economic and social stresses, particularly when combined with pre-existing toxic social norms and gender inequalities. In addition, strict public health measures – including orders to stay at home, social distancing and disrupted access to support services – led to an increase in gender-based violence, domestic homicides, violence against sexual and gender minorities, and child abuse.^9^

Sexual reproductive health and rights are generally an afterthought during emerging infectious disease outbreaks; the COVID-19 pandemic has also shown that these rights can become politicized and purposely limited.^9^ Also, pregnant individuals are at risk of exposure to antimicrobial resistance during pregnancy, abortion and childbirth, especially when these events take place in unsafe or unhygienic health-care settings.^10^ Females are generally prescribed more antibiotics and thus have more adverse drug events/reactions and antimicrobial resistance than males, which is both a sex- and gender-specific effect during emerging infectious disease outbreaks.^3^^,^^10^ Gender-specific roles and responsibilities, such as farming practices and health workers (for instance nurses, nurse aids and respiratory therapists), and/or norms that lead to gender-based violence, put women at risk of increased use of antibiotics, which can lead to antimicrobial resistance.^1^^,^^10^

## Addressing the gaps

Sex- and gender-specific approaches recognize that hormones mediate immune responses to infections and vaccinations, and that this is a key factor in the prevention and treatment of emerging infectious diseases.^11^ Health-care providers should understand these sex-mediated pharmacokinetic and pharmacodynamic changes due to hormonal status, to avoid over- or underdosing patients during treatment and/or vaccination for COVID-19, or any other emerging infectious diseases. Because these medications are scarce, understanding these sex differences is important to avoid adverse drug reactions and ensure rational use of the medications, which are necessary for critically ill, ventilated patients.^3^^,^^4^ Sex- and gender-specific factors play a major role in treatment outcomes – from pharmaceutical supply chain needs to treatment and sex-mediated pharmacodynamic effects on vaccination.^3^

Understanding sex and gender is central to learning from the COVID-19 outbreak and improving future pandemic preparedness. Recognizing the full scope of interactions between sex, gender and emerging infectious diseases will provide important insights into transmission patterns and strategies for outbreak prevention and control, will help reduce disease spread and will improve mortality and morbidity. Baseline, early recovery from outbreaks and post-disaster phases need complete gender analyses that include sexual and gender minorities. Doing so will help us better understand how emerging disease policies, programmes and interventions respond to or hinder the diverse needs of both sex and genders including sexual and gender minorities.^12^

Sex and gender- and hormonal disaggregated data, as well as gender-specific data (e.g. social roles and individual behaviours), complement epidemiological analyses. These data are essential for understanding the distributions of risk, infection and disease in the population, and the extent to which sex and gender play a role in stigma, discrimination, risk, treatment and clinical outcomes in pandemics.^1^^,^^2^^,^^5^^,^^6^^,^^12^ Since sex hormones alter gene expression, data collection, analysis and reporting must disaggregate, report and share data by sex, age and race/ethnicity to ensure that changes in hormonal and immune status are accounted for over the life cycle of individuals.^3^ Data that incorporate sex (for example, hormonal, anatomical, pharmacokinetic, pharmacodynamic) and gender factors provide a better understanding of what causes the observed virulence and outcome differences between sexes and genders and will help bring us closer to implementing equitable medical treatment and improve therapeutic choices.

The framework on sex, gender and emerging infectious diseases should be updated so it highlights all factors that affect mortality and morbidity differences that are sex- and gender-specific. Hormonal response, pharmacokinetics and pharmacodynamics as well as vertical and horizontal transmission factors of emerging infectious diseases complete the scope of sex-specific factors in terms of vulnerability, exposure and response to illness and treatment. Incorporating a more inclusive construct of gender and emphasizing the issue of gender-based violence will remind governments and policy-makers that gender-based violence plays a role in emergencies and will encourage them to develop responses to violence that are gender-specific and less focused on violence against women.^9^

Public health policies must create enabling environments and encourage the promotion of community health interventions that consider sex and gender vulnerabilities. Incorporating gender and sex differences as a priority at the local, national and global policy levels is crucial to fill the gaps on gender and infectious diseases in scientific research and public health intervention programmes and reduce emerging disease inequities during pandemics and epidemics.
